# Acoustic Emission and K-S Metric Entropy as Methods for Determining Mechanical Properties of Composite Materials

**DOI:** 10.3390/s21010145

**Published:** 2020-12-28

**Authors:** Lesław Kyzioł, Katarzyna Panasiuk, Grzegorz Hajdukiewicz, Krzysztof Dudzik

**Affiliations:** Faculty of Marine Engineering, Gdynia Maritime University, 81-225 Gdynia, Poland; l.kyziol@wm.umg.edu.pl (L.K.); g.hajdukiewicz@wm.umg.edu.pl (G.H.); k.dudzik@wm.umg.edu.pl (K.D.)

**Keywords:** composites, recycling, acoustic emission, K-S metric entropy, mechanical properties

## Abstract

Due to the unique properties of polymer composites, these materials are used in many industries, including shipbuilding (hulls of boats, yachts, motorboats, cutters, ship and cooling doors, pontoons and floats, torpedo tubes and missiles, protective shields, antenna masts, radar shields, and antennas, etc.). Modern measurement methods and tools allow to determine the properties of the composite material, already during its design. The article presents the use of the method of acoustic emission and Kolmogorov-Sinai (K-S) metric entropy to determine the mechanical properties of composites. The tested materials were polyester-glass laminate without additives and with a 10% content of polyester-glass waste. The changes taking place in the composite material during loading were visualized using a piezoelectric sensor used in the acoustic emission method. Thanks to the analysis of the RMS parameter (root mean square of the acoustic emission signal), it is possible to determine the range of stresses at which significant changes occur in the material in terms of its use as a construction material. In the K-S entropy method, an important measuring tool is the extensometer, namely the displacement sensor built into it. The results obtained during the static tensile test with the use of an extensometer allow them to be used to calculate the K-S metric entropy. Many materials, including composite materials, do not have a yield point. In principle, there are no methods for determining the transition of a material from elastic to plastic phase. The authors showed that, with the use of a modern testing machine and very high-quality instrumentation to record measurement data using the Kolmogorov-Sinai (K-S) metric entropy method and the acoustic emission (AE) method, it is possible to determine the material transition from elastic to plastic phase. Determining the yield strength of composite materials is extremely important information when designing a structure.

## 1. Introduction

In recent years, an increase in the use of glass fiber reinforced polyester laminates has been observed in many industrial branches, including shipbuilding [1,2,3]. The use of composite materials in shipbuilding results from their specific properties, such as resistance to rotting in the sea water environment, as well as corrosion and the effects of chemicals. These materials are characterized by high resistance to fatigue loads, especially typical for the operation of the hull or elements of the marine propulsion system on a sea wave [1,2,3]. Ship propulsion systems require specific technical solutions, the features of which result not only from engineering practice, but mainly from the requirements of classification societies [2,3,4]. Polyester laminates in conditions of salinity with air mist show high resistance to corrosion. They are also used for bearing and sealing propeller shafts. Laminates are used for sealing and bearing the rudder stocks, they are also used for such elements as deck crane bearings, washers for the sliding hatch cover, in mooring systems and in hydraulic devices for operating pressures ranging from 25 to 100 MPa [5].

Due to their anisotropic properties, composite materials constantly ask designers to define the parameters of mechanical properties with high precision. One such problem is determining the transition of a material from elastic to plastic. Such a problem does not exist in elastically plastic materials. Currently, the researcher has more and more perfect machinery and equipment facilities, which creates new opportunities for a more detailed understanding of the properties of anisotropic materials, which are undoubtedly polymer composites.

Having the appropriate equipment facilities, the authors set themselves the goal of determining the yield point of the composite material with the greatest possible accuracy. The knowledge of this material parameter allows for greater use/effort of the material, which is of great importance when designing the structure. For composite materials, the standards do not provide for the determination/knowledge of the yield point. For this reason, the calculations of composite-based structures are most often underestimated. One of the methods allowing to determine the yield point of the tested polymer composites is the method of acoustic emission. Basically, this method, classified as a non-destructive method, is used to monitor the process of damage to materials (structures). Monitoring can be carried out under both operational and laboratory conditions. The tested object is subjected to a load that is recorded. The applied amount of force cannot lead to the complete destruction of the structure [6]. Acoustic emission (AE) is a phenomenon in which elastic waves are generated, forced by stresses resulting from structural changes (fiber cracking, matrix cracking, etc.) in solids. The frequency of AE waves in the range from a few kHz to 1 MHz is detected on the structure surface by piezoelectric sensors that convert the strain energy into an electrical signal [6,7,8,9,10,11,12,13,14,15,16,17].

The AE method is especially used to test fiber-reinforced polymer structures [6,7,9,12,13,14,15,16,17]. The AE bending test described in [6] contributed to the development of the AE signal identification methodology. This resulted in the identification of three characteristic time intervals: initiating damage (DI), cumulative damage (DA), and localization damage (DL) [6].

The static tensile test of bars reinforced with polymer fibers, during which various damages were registered (delamination, matrix cracking, fiber detachment and cracking)-contributed to the determination of the size and frequency of each signal [13].

The paper [14] describes the mechanical damage of a thick laminated carbon-epoxy composite subjected to static and fatigue tests. Three types of signals were related to three failure modes (matrix cracking, delamination, fiber breakage). Parameters such as amplitude, duration and energy were analyzed [14]. The papers [15,16,17] describe the use of acoustic emission parameters to identify the type of damage.

Various mathematical methods are used to study the properties of materials. Modern measurement tools and methods ensure their very high accuracy and obtain large data sets. In order to organize these sets and obtain results, one can use the methods used, among others, in mathematical statistics. In search of deviations of experimental data from their mean values, an analysis is performed, and the interpretation of measurement errors and distortions is made. The end result is specific procedures which include, among others, limits, frames, and divisions of numerical values to which further experimental data should be assigned. In engineering practice, there are numerous obstacles resulting from a significant dispersion of mean values. Spreading measurement data across studies can take complex forms, and it can be difficult to identify a single parameter to control a particular process. Examples are the graphs of the static tensile test, on the basis of which the internal dynamics of the material deformation process cannot be observed. The dynamics of the tensile deformation process of the sample in the form of chaotically arranged measurement points (especially in the elastic range of the material) can only be observed by significantly enlarging a part of the graph [18,19,20,21,22].

The occurrence of deterministic chaos in experimental data sets, characterized by the behaviour of materials under given loads in real structures or in laboratory conditions on strength machines, has not yet been sufficiently described [22].

Earlier tests carried out on materials showing a clear yield point showed a high agreement of the yield strength values obtained in accordance with the standard and the specified K-S entropy method [18,19,20,21,22,23]. On the basis of a series of laboratory tests carried out by the authors of the study for isotropic and anisotropic materials, it was assumed that the application of the K-S method allows to determine the transition of the material from the elastic to the plastic phase. The method of visualizing the changes in the dynamics of data obtained from strength tests in laboratories or from measurements on real objects may be of great importance for the future of design and modelling of composite materials.

In the article, on the example of a polyester-glass composite with and without recyclate, research was undertaken to determine the yield point. For this purpose, the acoustic emission (AE) method and the K-S metric entropy method were used.

## 2. Materials and Methods

The glass polyester recyclate used for testing is a fragment of the main deck sourced from one of the ships. This scrap was pre-crushed using a mechanical hammer, and then ground in a dedicated plastic waste processing station. Crushed this way, the material was screened on sieves to obtain various fractions. A 1.2 mm sieve mesh was used. The 1.2 mm granulate obtained is referred to as the recyclate and was used as the filler of a newly produced glass polyester composite matrix.

Producing a proper grain-sized recyclate was followed by producing a composite on the basis of resin, glass mat used as reinforcement, and the recyclate-used as the filler. Manual contact lamination was used to produce the new composite. Polyester, structural, orthophthalic, rigid POLIMAL 109A resin manufactured by Organika-Sarzyna S.A. and a hardening and booster agent were used as matrix for all materials produced. Two different composite materials with different weight additives were produced: basic material without recyclate (pure), material with a 10% recyclate content [24,25,26,27,28,29]. The contents of% by weight of the reinforcement, matrix and recyclate are presented in Table 1.

**Figure 1:**
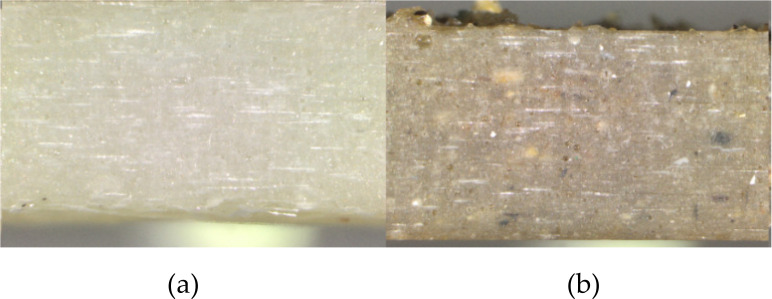
Cross-sections of composite materials with recyclate content of 1.2 mm granulation and percentage content: (**a**) 0%, (**b**) 10%, magnification ×3 (confocal microscope).

Prepared materials are shown on Figure 1.

**Figure 2:**
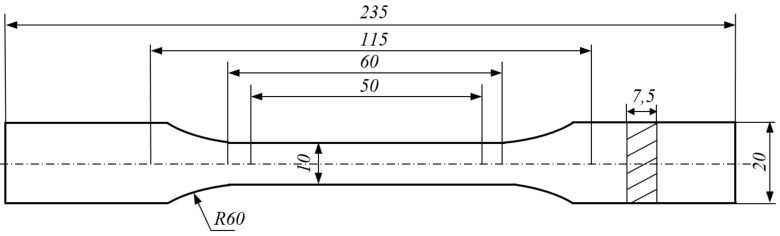
Dimensions of samples for static tensile test in accordance with PN-EN ISO 527-4_2000P.

The mechanical properties of composites with the addition of glass polyester recyclate were determined according to PN-EN ISO 527-4_2000P. For this purpose, each of the two composites was used to prepare samples for static tensile testing. The shape and dimensions of the samples are shown in Figure 2.

The static tensile test of the samples from the tested composites was carried out on a universal testing machine with hydraulic drive, type MPMD P10B, with TestXpert II software, version 3.61. by Zwick & Roell with the Epsilon model 3542 extensometer (Epsilon Technology Corp., Jackson, WY, USA). The tests were carried out on tests at ambient temperature (22 °C) at the strain rate equal to 2 × 10^−5^ s^−1^.

### 2.1. Acoustic Emission Method

**Figure 3:**
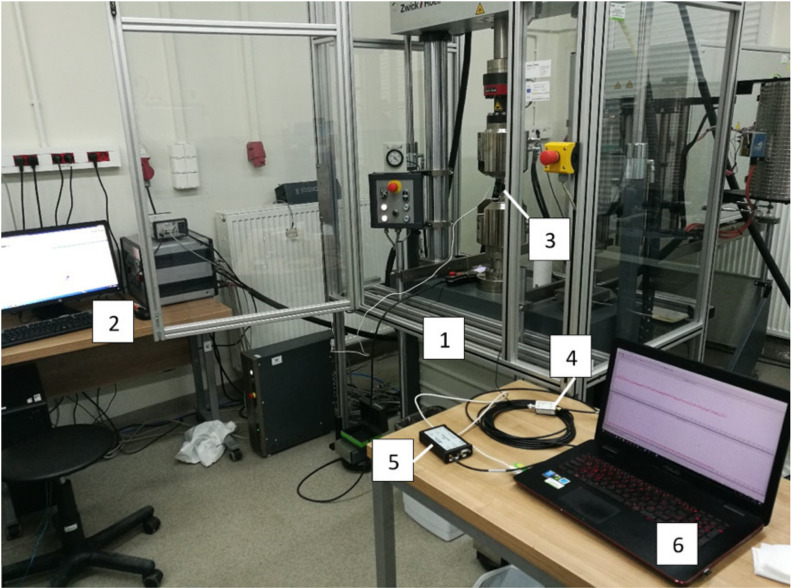
General view of laboratory stand: 1–tensile stress machine, 2–tensile stress machine computer, 3–AE sensor, 4–preamplifier, 5–AE recorder, 6–AE computer [30].

The research was carried out at the universal hydraulic testing machine Zwick&Roell MPMD P10B with TestXpert II software. Additionally, Epsilon 3542 extensometer was used for measuring elongation during test. For monitoring tensile test of chosen specimens Physical Acoustics Company (Physical Acoustic Corporation, 195 Clarksville Rd, Princeton Junction, NJ, USA) acoustic emission system was used. A general view of the laboratory stand is presented in Figure 3 [30].

**Figure 4:**
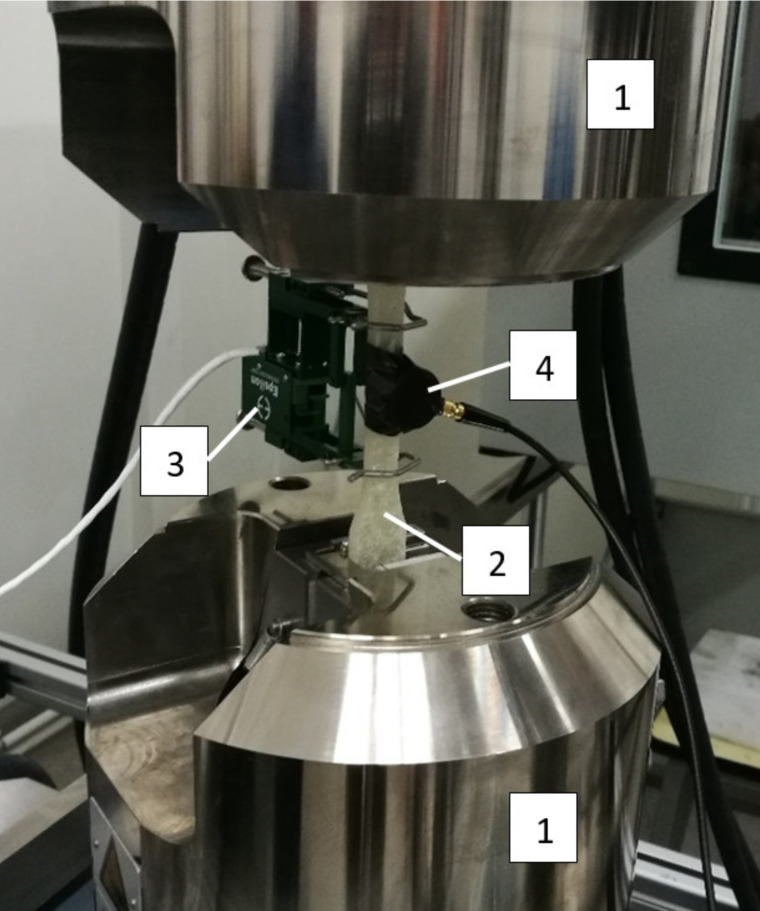
View of specimen fixed into tensile testing machine grips: 1–tensile testing machine grips, 2–specimen, 3–extensometer, 4–AE sensor [30].

A view of the specimen fixed into tensile testing machine grips during research, with AE sensor and extensometer, is shown in Figure 4.

Research AE was performed using set consisted of a single channel recorder USB AE Node (Physical Acoustic Corporation, Princeton Junction, NJ, USA), type 1283 with bandpass 20 kHz–1 MHz, preamplifier with bandpass 75 kHz–1.1 MHz, AE-Sensor vs. 150 M (with a frequency range of 100–450 kHz), computer with AE Win for USB Version E5.30 software for recording and analysing AE data.

Between the sensor and a surface of the specimen a coupling fluid was used. AE Sensor was fixed to specimen by elastic tape.

### 2.2. Metric Entropy Method

**Figure 5:**
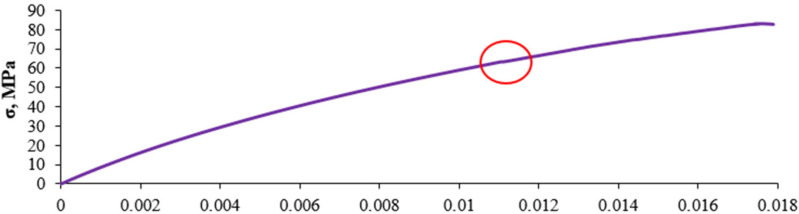
An example graph of a static test on stretching the composite with a 10% recyclate content.

**Figure 6:**
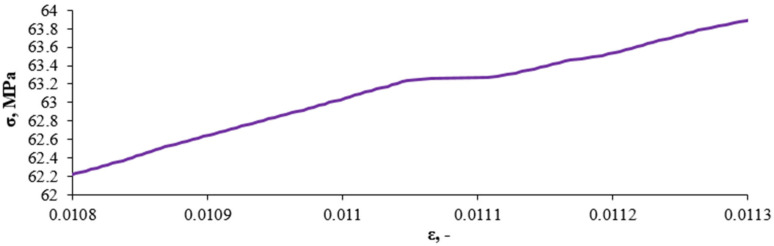
Chaotic arrangement of the measurement points.

Figure 5 shows an example graph of a static test on stretching the composite with a 10% recyclate content. This kind of a graph doesn’t present the inside dynamics of the process of distorting materials. The chaotic arrangement of the measurement points is presented on the Figure 6 by means of the consecutive enlargements of the fragments marked of the graph on the Figure 5.

Figure 6 shows a fragment of a static tensile test of the composite containing 10% of recyclate.

The most important factor that influences the precision of a test concerns the type of a specimen that generates measurement distortion, depending on the starting point state of a specimen’s material and the conditions of its fixing. Anisotropy and distribution of stresses within the material, the structure and material’s crystallographic defects distort the homogeneity of the stresses’ distribution and strains in the specimen’s measurement field. They lead to the development of local fields of plastic strains also when treated with small forces, which don’t exceed the elastic limit. Resolution, in other words, the precision of the measurement, is defined on the basis of the noise measurement. Other factors that influence the test’s results are related to one another in a system machine-specimen, that is controlled in a loop of feedback. The behaviour of this system depends on hydraulic system’s quality and efficiency as well as the characteristics of a specimen being tested. Parameters of the feedback between the specimen and the machine must be regulated according to the specimen’s predicted characteristics. Any distortions in the system machine-specimen, are visible especially when the characteristic of a certain material changes, i.e., during the transition from an elastic into a plastic range, when transition into plastic strains occurs and rigidity of the measured system undergoes a sudden decrease. Damage of the specimen initiates a local process, which has got an effective impact on the dynamic characteristic of the whole system. During this transition, we can observe an increase of force error in relation to the applied force, but it is not only due to the measurement precision, but the total effect of the measurement track’s resolution, the efficiency of hydraulic system, the setting of the steering, and the degree of changes of the material’s characteristics.

Classic thermodynamic entropy entails the flow energy from large to small scale [31,32,33,34]. According to the second law of thermodynamics, the total entropy of an isolated system can never decrease over time, and will thus always approach positive values. A statistical interpretation of the second law of thermodynamics was coined by L. Boltzmann. He demonstrated that macroscopic state entropy *S* is proportional to the thermodynamic probability of this state. This interrelation was expressed by formula:

(1) S=k lnW

This means that the dynamics of a system leads to the formation of increasingly probable states as it approaches the maximum value of thermodynamic probability. K-S metric entropy [22,31,32,33,34] joins opposite-direction energy flow, i.e., transitions from small-scale to large-scale dispersion. Energy dispersion is never a continuous process. As a dynamic notion, K-S entropy is the “entropy of a time unit” [22], and is therefore non-negative, but can both increase and decrease. The relationship between statistical mechanics and the chaos theory reflects the notion of K-S metric entropy, which was introduced by Kolmogorov [35].

The essence of metric entropy is that it is dynamic by nature, as it describes system movement typical for chaotic processes [22,31,32,33,34]. Metric entropy is a value that measures the instability of the dynamics of a system, i.e., expresses a method for the numerical description of chaos.

Works [18,19,20,21,22,23] present an overview of a measurement collection and a diagram for calculating K-S entropy for successive positions of measurement collections containing an n number of items, relative to measurement data.

For a discrete probability distribution, K-S metric entropy [35,36] is described using the following formula:

(2) $$S=-\sum_{i=1}^{N}p_{i}\ln p_{i}$$

where:

*N* denotes number of partitions, into which the set of all possible results was divided and *p*_*i*_ is the probability of occurrence of results in the ith partition (according to *p* ln *p* = 0, if *p* = 0).

If the partitions are equally probable, which means that *p*_*i*_ = 1/*N* for all *i*, then entropy is defined by *S* = ln *N* and assumes its maximum value. However, if the results are known for specific partitions, then entropy will assume its minimum value of *S* = 0, since *p*_*i*_ = 1. The conditional extremes of the entropy function were determined using the Lagrange multiplier method [35,36].

The K-S metric entropy method assumes that the qualitative changes which take place at the structural threshold separating the elastic state from the plastic state correspond to a specific measurement point. Energy dissipation takes place in the system, and the deterministic chaos of data related to this phenomenon causes the variability of entropy.

In this method, it is essential to prepare the input data used to calculate metric entropy correctly. In the sample stretching process, the tester must record the stress and the strain, and a column of ε σ quotients must be created at the same time. The sequence of successive strains, stresses and ε σ quotient remainders is formed by deducting successive rows. As the first derivative of the time sequence, it reflects the local dynamic of measurement data. For input data prepared in this manner, a measurement collection occupies successive positions in a column of numbers, shifting by a single row every entropy calculation step. Determined this way, entropy is them recorded on a diagram.

The minimum value of metric entropy in the vicinity of the passage from elastic to plastic state is marked by the point that separates individual states of the process. The value of stress corresponding to the passage of the tested material from its elastic to its plastic state is determined on the basis of the “critical” point [18,19,20,21,22,23].

To determine the “critical” point which corresponds to the transition of the material from one state to another, we need to have a declared number of constituents *k* in the measurement collection, and a number of sub-partitions *N*, into which the measurement collection will be divided. Based on the data from tensile testing, the effect of *k* and *N* values on the shape of the entropy diagram and the position of the minimum value relative to the tensile curve were analyzed. Measurement data includes 1000 to 2000 points in the elastic scope. The *N* number was adopted according to a formula applied in mathematical statistics in the construction of histograms: *N* ≤ 5 *log k* [18,19,20,21,22,23].

An analysis of determination of passage from the elastic state to the plastic state, applying the “minimum entropy” method on tested materials, has indicated that the correct selection of the k number of constituents of the measurement collection and the *N* number of sub-partitions, is key to drawing the K-S metric entropy fluctuation diagram, marking a clear minimum which confirms the passage from one state to another. Correctly prepared data can be then entered to calculation software [18,19,20,21,22,23].

## 3. Results

Table 2 shows all results of a static tensile test of samples of composite materials tested are given.

Table 2 presents the mean values for two composite materials. Three samples were taken from each material for the static tensile tests.

**Figure 7:**
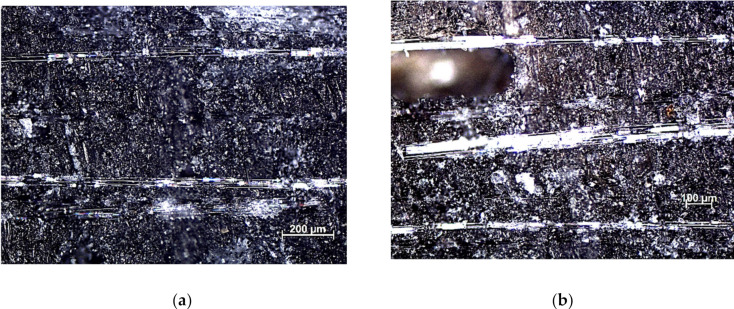
Composite structures: (**a**) without recyclate, (**b**) 10% of recyclate, magnification ×100.

The study of the structure of the composites was carried out on samples by optical microscope (Axiovert 25, Carl Zeiss AG, Oberkochen, Germany). The samples were cut across (in thickness) and the surface was analyzed. Figure 7 shows the structures of composite materials.

In general, the mechanical properties of the composite depend greatly on the ability of the matrix and filler to adhere well to each other. The structures of composites presented in Figure 7 slightly reflect their mechanical properties. In the case of a material without recyclate (Figure 7a), evenly distributed glass fibers are visible, which results in high adhesion of the resin to the glass fiber. This guarantees a high physical contact of the fiber with the polyester, which results in high strength properties of the composite.

**Figure 8:**
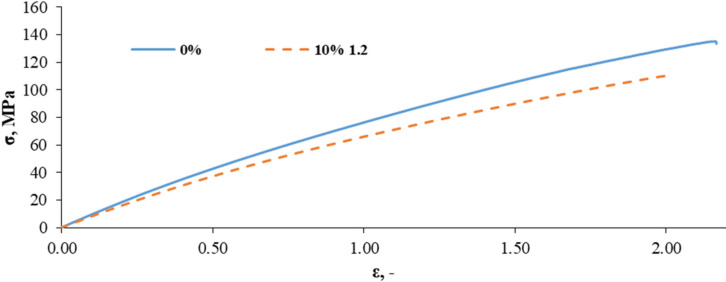
Results of the static tensile test for composite samples.

The introduction of polyester-glass recyclate to composites reduced their tensile strength. In the structure of the composite with 10% recyclate content (Figure 7b), air pores and areas in the form of recyclate clusters are visible. The result is a reduction in the strength properties of the material. Figure 8 shows the results of a static tensile test for composite materials.

A static tensile test carried out on composite samples without recyclate content and with 10% recyclate content pointed to a clear impact of recyclate content on the mechanical properties of the composites tested. The results of the static test pointed to a deterioration of the mechanical properties of the composite, proportional to the increase in recyclate content.

Obtained results from the static tensile test of composite materials with the addition of recyclate served as the input material for the application of metric entropy K-S to determine the yield point of the composite materials tested.

Entropy is calculated using stress and strain data measured in the process of stretching the samples. In this study, the samples were stretched by applying fixed force (fixed movement speed is also possible). The resultant diagram of K-S entropy in the function of time is closely related to the stress diagram, which also entails the function of time.

Before proceeding to the primary calculations, the number of n-elements of the data collection divided into an *N* number of partitions must be determined. The ultimate *n* and *N* values are heuristically determined. The correct selection of n-elements and N-partitions affects the correctness of the problem to be solved, and this on the correctness and transparency of the resultant diagrams. Correctly solved, the problem can be used to point the local minimum of K-S entropy, which indicates the passage from the elastic state to the plastic state.

To calculate metric entropy, deformation data is used during a static tensile test. In the example below the first 60 deformation results were used. (Table 3). Their minimum and maximum for the selected 60 values (n-elements) have been established (min = 0.00002711, max = 0.00014899). The minimum value was subtracted from the maximum value and this value was divided into 4 (*N*—number of subsets). Then this value (0.00003046) was added to the minimum value, thus obtaining the first subset, subsequent subsets were created while adding the calculated value in sequence. Then the probability of finding the deformation value in the next subset is determined. The next step is to calculate the logarithm of probability and multiply the probability and logarithm of probability. Substitution of the obtained values, using the K-S entropy formula, allows calculating the values for 30 deformation values, i.e., half.

(3) $$S=-\sum_{i=1}^{N}p_{i}\ln p_{i}=-(-0.3535-0.3380-0.3466-0.3466)=1.3847$$

**Figure 9:**
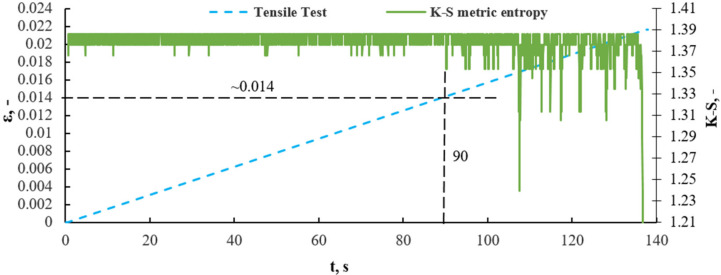
A metric entropy diagram applied on a strain diagram in the function of time, for a sample of composite without recyclate.

This is exemplified by a study [18,19,20,21,22,23], in which the application of correct procedures and an authorial program yielded correct results. Figure 9 presents a metric entropy diagram applied on a strain diagram in the function of time for a sample of composite without recyclate.

**Figure 10:**
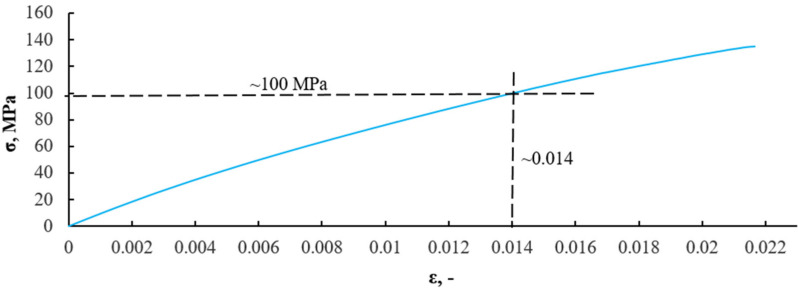
Determination of stress value on a composite without recyclate sample corresponding to strain ε = 0.014 (90 s of time in Figure 9).

In the 90 s of time, the lowest entropy value corresponded to strain *ε* = 0.014. At this point, according to the K-S metric entropy theory, structural changes occur in the material. Analyzing previous test results for isotropic materials with a clear yield point [18,19,20,21], the mathematical statistics method showed exactly the same point as the yield point on the tensile graph. It can be stated that irreversible changes occur in the material at this point and can be defined as the transition from the elastic to plastic phase in the composite material. Figure 10 presents the diagram ε-σ with the strain of 0.017 marked and corresponding to a stress of 116 MPa.

**Figure 11:**
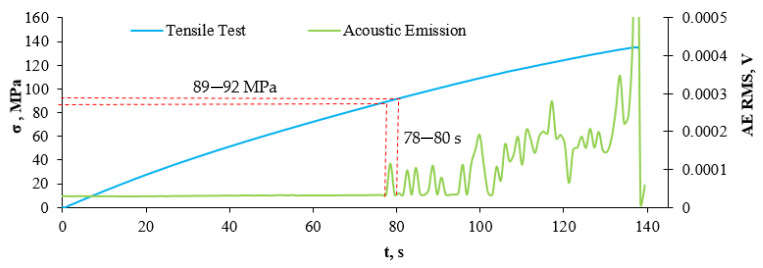
Graph of the value of the effective acoustic emission signal as a function of time, plotted on the stress-strain graph for a composite without recyclate.

After transferring the dependence of strain and K-S entropy from time, the strain values in the strain stress diagram, we obtain the stress value for the transition from the elastic (linear) phase to the nonlinear phase. In the case of the material without recyclate content, the structural change, defined as the transition from the elastic phase to the non-linear range phase, occurred at a stress value of about 116 MPa and a strain of about 0.014. Figure 11 presents a graph of the value of the effective acoustic emission signal as a function of time, plotted on the stress–strain graph for a composite without recyclate.

**Figure 12:**
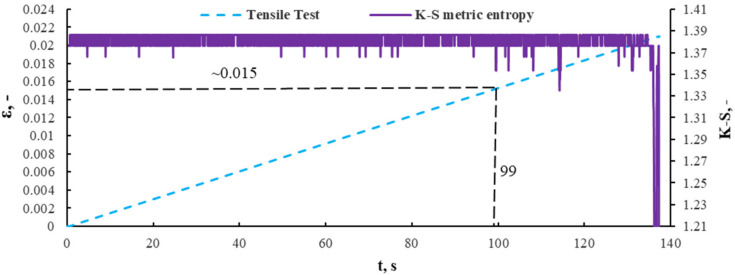
A metric entropy diagram applied on a strain diagram in the function of time, for a composite sample with a 10% recyclate content and ≤1.2 mm in mesh size.

The diagram presented in Figure 11 shows that the beginning of structural changes in the material occurred in the range of 78–80 s, which corresponded to the stress of 89–92 MPa. The calculations carried out by the K-S method showed that it corresponds to the transition of the material from the elastic to the plastic phase. Figure 12 presents a metric entropy diagram applied on a strain diagram in the function of time, for a composite sample with a 10% recyclate content and 1.2 mm in mesh size.

**Figure 13:**
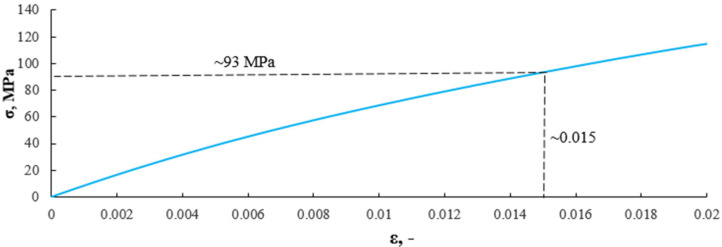
Determination of stress value on a composite sample with a 10% recyclate content and 1.2 mm in grain size corresponding to strain ε = 0.015 (99 s of time in Figure 12).

In the 99 s of time, the lowest entropy value corresponded to strain *ε * = 0.015. For a material with a 10% recyclate content, the deformation, with a significant decrease in entropy, metric K-S, was 1.34. Structural changes have occurred in the material with less deformation than in the material without recyclate content, which results directly from the mechanical properties of the material. Figure 13 presents the diagram σ(ε) with the strain of 0.015 marked and corresponding to a stress of 82 MPa.

**Figure 14:**
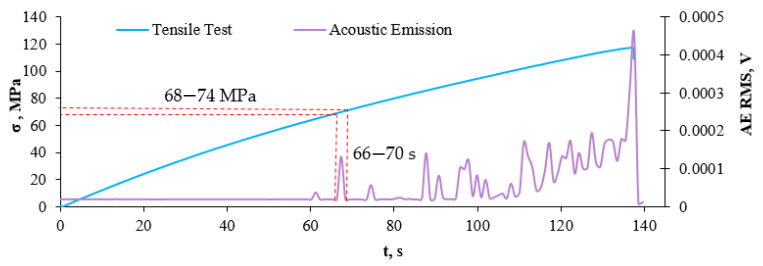
A metric entropy diagram applied on a strain diagram in the function of time, for a composite sample with 10% recyclate content.

As a result of the transfer of the dependence of strain and entropy K-S on the time of the strain value on the stress strain graph, we obtain the stress value for the transition from the elastic phase to the non-linear range phase. In the case of a 10% recyclate content, a structural change occurred, defined as the transition from the elastic to the non-linear range phase at a stress of about 93 MPa and a deformation of about 0.015. Figure 14 presents a graph of the value of the effective acoustic emission signal as a function of time, plotted on the stress–strain graph for a composite with 10% content of recyclate.

Based on the results obtained by the AE method, a significant increase in the RMS value of the signal appeared in 68–74 s, which corresponds to a stress of 66–70 MPa. The values of stresses obtained by the method of K-S metric entropy and acoustic emission are similar, which indicates the occurrence of a qualitative change in the material, i.e., a transition from elastic to non-linear range phase. Table 4 presents a summary of the results obtained using the K-S and AE method.

The results obtained by the K-S entropy method and the acoustic emission (AE) method show good agreement. Interpretation of the charts presented in Figure 8, Figure 9, Figure 10, Figure 11, Figure 12, Figure 13 and Figure 14 allows for the assessment of qualitative changes in the material. In particular, the transition of the composite without recyclate from the elastic phase to the nonlinear phase, determined by the K-S method, took place at a stress of approx. 100 MPa and the AE method in the range of 89–92 MPa. In the case of a composite with the addition of 10% recyclate, the appropriately determined stress values by the K-S method are 93 MPa, and by the AE method are 68–74 MPa. Characteristically, the emission shows results that are on average 15 MPa lower than the metric entropy. In each analyzed case, using the acoustic emission method, changes are detected earlier and at lower stress values. The K-S metric entropy method is based on elongation data (in the case of constant force growth rate) and it is a mathematical statistical method. Metric entropy is a value that measures the instability of the dynamics of a system, i.e., it expresses a method of numerically describing chaos. This means that the metric entropy method, using the machine-sample relationship, allows you to determine the point (local minimum K-S metric entropy) at which there is chaos in the obtained measurements and at the same time the transition from elastic to nonlinear phase. The acoustic emission analyzes the transient elastic waves resulting from the processes taking place in the composite during loading, such as the cracking of fibers, matrix, etc. The shift in time results from the fact that the acoustic emission ‘detects’ changes in the material before the elastic to nonlinear. The AE method is a very accurate method that allows you to directly record test results. However, the K-S metric entropy method can also serve as a tool to assist in the description of material changes under stress. This method should be improved and successfully used in material testing.

## 4. Discussion

The results of the research showed that the addition of a larger number of granules (over 10%) has an unfavorable effect on composites, as it significantly reduces their strength. The mechanical strength of the composite depends largely on the ability of the matrix and filler and their good adhesion to each other. Basically, the addition of milled polyester/glass granules to composites resulted in a reduction in tensile strength due to poor interfacial adhesion of the granule and matrix particles.

Due to their granular nature, higher contents of granules result in a rougher surface with numerous microcracks, making the composite surface rougher. Research has shown that composites containing granules have a rough surface with many microcracks (Figure 7a,b). The surface of the composite without granules shows a smooth surface with few microcracks. The smooth surface is associated with strong intermolecular bonds, which in turn leads to an increase in tensile strength. The microstructure tests showed that in the tensile test, stronger adhesion between the matrix granules was observed, which gave the composite higher tensile strength.

An analysis of the diagrams presented in Figure 9, Figure 10, Figure 12 and Figure 13 points to a close relationship and regularity between metric entropy diagrams and tensile diagrams. Applying the values of this strain on the diagram σ(ε) (Figure 10), we can define the value of stress (100 MPa). Analogously, in Figure 13, minimum entropy value was determined for a sample with a 10% recyclate content and 1.2 mm in grain size, for which the strain was ε = 0.015. The values of this strain correspond to a stress of σ = 93 MPa (Figure 13).

The tensile strength of the composite without recyclate is 135 MPa (Table 2), and the yield point is in the range of 89–100 MPa. In the case of a composite with 10% recyclate content with a tensile strength of 110 MPa, the yield point is in the range of 74–93 MPa. This requires clarifying the research methods. Research results indicate that research should be continued and research methods improved.

In addition to the decrease in tensile strength, the value of the composite elasticity modulus also decreases with the increase in the granulate content. Due to the lower content of granules, the bonds between the granulate particles and the polyester matrix have a positive effect on their interfacial adhesion and prevent damage to the composite.

Two research methods presented in the paper, aimed at determining the transition of a material from the elastic phase to the nonlinearity phase, have shown their practical usefulness. While the AE method is commonly used in material research, the K-S metric entropy method requires elaboration. The difference between the results obtained when using these methods is approx. 10–20%. It is small, and so, when calculating marine structures made of composite materials, the safety factor should be taken into account, the value of which is within a large range. The accuracy of the results obtained may not be of great importance. The use of modern instrumentation and new calculation methods creates great opportunities for detailed values of material parameters. It is of great importance considering the increasing use of composite materials for very complex and complicated ship structures.

## 5. Conclusions

1. The use of modern measuring devices and calculation methods enables the determination of material parameters with extremely high accuracy.
2. The method of metric entropy (K-S and acoustic emission AE) allows to determine the transition of the material from elastic to non-linear range phase.
3. K-S metric entropy and acoustic emission method are an excellent engineering tool useful for determining material constants.
4. Both methods create new possibilities for testing materials, especially in terms of fatigue strength.
5. Research should be continued in order to improve research methods of materials due to more and more modern test stands and measuring instrumentation.

## Figures and Tables

**Table 1:** The contents of% by weight of the reinforcement, matrix and recyclate in prepared materials.

	Number of Layers of Reinforcement	Matrix, % by Weight	Resin, % by Weight	Recyclate, % by Weight
0%, without recyclate	12	40	60	0
10% recyclate, ≤1.2 mm granulation	12	29	61	10

**Table 2:** Mean values of a static tensile strength test carried out on composite materials obtained from three samples taken from each material.

Recycled Content, % Weight, mm		*E*, MPa	$R_{m}$, MPa	ε, -
**0**	**Mean Value**	8982	135	0.0216
	**Standard Deviation**	0.53	0.87	0.0057
**10%, 1.2**	**Mean Value**	7830	110	0.0201
	**Standard Deviation**	0	0.39	0.0057

**Table 3:** Procedure of calculating K-S metric entropy.

	I	II	III	IV
min	0.00002711	0.00005758	0.00008805	0.00011852
max	>0.00005758	>0.00008805	>0.00011852	≥0.00014899
	0.00002711	0.00005880	0.00008959	0.00012039
	0.00002902	0.00006131	0.00009106	0.00012227
	0.00003102	0.00006385	0.00009264	0.00012416
	0.00003303	0.00006645	0.00009452	0.00012596
	0.00003495	0.00006902	0.00009647	0.00012759
	0.00003673	0.00007160	0.00009849	0.00012943
	0.00003847	0.00007398	0.00010057	0.00013141
	0.00004022	0.00007624	0.00010277	0.00013364
	0.00004198	0.00007846	0.00010498	0.00013593
	0.00004370	0.00008050	0.00010739	0.00013817
	0.00004553	0.00008252	0.00010979	0.00013997
	0.00004745	0.00008415	0.00011209	0.00014220
	0.00004961	0.00008603	0.00011432	0.00014466
	0.00005190	0.00008779	0.00011646	0.00014675
	0.00005407		0.00011848	0.00014899
	0.00005637			
*p_i_*	0.27	0.23	0.25	0.25
ln *p_i_*	−1.30933332	−1.46967597	−1.38629436	−1.38629436
*p_i_*ln *p_i_*	−0.35352000	−0.33802547	−0.34657359	−0.34657359
*S*	1.38469265			

**Table 4:** Summary of results for analysed samples obtained with the AE method and the K-S metric entropy.

Recycled Content, % Weight, mm	Transition from Elastic to Nonlinearity by the Method of K-S Metric Entropy, *R_K-S_,* MPa	Transition from Elastic to Nonlinearity (Range) by AE Method, *R_AE_*, MPa
0%	~100 MPa	89–92 MPa
10%, ≤1.2 mm	~93 MPa	68–74 MPa
